# Structure–functional relationship of cellular retinoic acid-binding proteins I and II interacting with natural and synthetic ligands

**DOI:** 10.1107/S2059798320015247

**Published:** 2021-02-05

**Authors:** Charles W. E. Tomlinson, Katy A. S. Cornish, Andrew Whiting, Ehmke Pohl

**Affiliations:** a Department of Chemistry, Durham University, Lower Mountjoy, South Road, Durham DH1 3LE, United Kingdom; b Department of Biosciences, Durham University, Upper Mountjoy, South Road, Durham DH1 3LE, United Kingdom

**Keywords:** retinoids, synthetic retinoids, retinoic acid-binding protein, CRABPI, CRABPII, ligand binding

## Abstract

A detailed understanding of the interactions between small-molecule ligands and their proposed binding targets is of the utmost importance for modern drug-development programs. Cellular retinoic acid-binding proteins I and II (CRABPI and CRABPII) facilitate a number of vital retinoid signalling pathways in mammalian cells and offer a gateway to manipulation of signalling that could potentially reduce phenotypes in serious diseases, including cancer and neurodegeneration. Although structurally very similar, the two proteins possess distinctly different biological functions, with their signalling influence being exerted through both genomic and nongenomic pathways. In this article, crystal structures are presented of the L29C mutant of *Homo sapiens* CRABPI in complex with naturally occurring fatty acids (1.64 Å resolution) and with the synthetic retinoid DC645 (2.41 Å resolution), and of CRABPII in complex with the ligands DC479 (1.80 Å resolution) and DC645 (1.71 Å resolution). DC645 and DC479 are two potential drug compounds identified in a recent synthetic retinoid development program. In particular, DC645 has recently been shown to have disease-modifying capabilities in neurodegenerative disease models by activating both genomic and nongenomic signalling pathways. These co-crystal structures demonstrate a canonical binding behaviour akin to that exhibited with all-*trans*-retinoic acid and help to explain how the compounds are able to exert an influence on part of the retinoid signalling cascade.

## Abstract 2

Structures of *Homo sapiens* cellular retinoic acid-binding proteins I and II in the presence of natural and synthetic ligands are presented, demonstrating canonical binding activity for these new molecules and relating the structure and function of these novel retinoid signalling modulators.

## 1. Introduction   

The cellular retinoic acid-binding proteins CRABPI and CRABPII act as gatekeepers and facilitators for the passage of all-*trans*-retinoic acid (and related derivatives) through the cytoplasm to access retinoic acid receptors (RARs) in the nucleus. This journey is of eminent importance, as retinoid signalling has a huge impact on the growth and development of mammalian organisms throughout their lifetimes, with a particular focus on embryonic development. Beyond this, the general retinoid signalling system and vitamin A-utilization pathways are relied upon for vision and skin health, and have been implicated in the treatment of various cancers and neurodegenerative diseases (Moon *et al.*, 1979 ▸; Cowan *et al.*, 2017 ▸; Sahin *et al.*, 2005 ▸; Katz *et al.*, 1999 ▸; Khatib *et al.*, 2019 ▸, 2020 ▸).

CRABPI shares high sequence identity (77%) with CRABPII but, unlike its sister protein, does not contain the appropriate residues to form a nuclear localization sequence (NLS). As a result, it localizes entirely into the cytoplasm and carries out the role of buffering retinoid concentrations within the cell, aiding in the degradation of retinoids via cytochrome P450s (Ross & Zolfaghari, 2011 ▸). Additionally, CRABPI can modulate several putative nongenomic pathways: the ERK1/2 kinase pathway is dose-dependently modified by the complex of CRABPI with all-*trans*-retinoic acid (ATRA) *via* sequential interactions with Raf, MEK and ERK kinases (Park *et al.*, 2019 ▸; Persaud *et al.*, 2013 ▸), and in mice the calmodulin-dependent protein kinase pathway is modified by reduced autophosphorylation of CaMKII in the presence of CRABPI (Park *et al.*, 2018 ▸).

In contrast, CRABPII acts as flagship for ATRA transport in mammalian cells and is capable of nuclear localization owing to a ‘noncanonical nuclear localization sequence’ composed of three residues, Lys20, Arg29 and Lys30, which are located spatially together across the helical cap when the protein is ligand-bound but are separated by several residues in the primary sequence (Sessler & Noy, 2005 ▸). This allows CRABPII to ferry retinoic acid from the cytoplasm into the nucleus, where it is further transferred to the RAR/RXR nuclear receptors (Budhu *et al.*, 2001 ▸). The retinoic acid-binding ability of both CRABPI and CRABPII is centred on a hydrophobic pocket created by an orthogonal β-sheet fold and capped with a pair of antiparallel α-helices formed from residues 15–35 partway into the sequence (Kleywegt *et al.*, 1994 ▸). The pocket contains, at its most buried end, a trio of residues (Arg112, Arg132 and Tyr134 in CRABPI; Arg112, Arg133 and Tyr135 in CRABPII) that are responsible for binding to the carboxylic acid moiety of ATRA, usually in concert with a water molecule that is conserved in all structures with similarly binding ligands. The rest of the pocket is lined with hydrophobic residues that create a suitable environment for the unsaturated, fatty-acid-like portion of ATRA and, when unoccupied, contains a network of water molecules that support the fold (Vaezeslami *et al.*, 2008 ▸). The hydrophobic contribution to the binding energy is considerable, as even when other elements of the binding mode are disturbed by mutation, general binding efficiency is maintained (Vasileiou *et al.*, 2009 ▸). The structure of CRABPII has previously been determined in both ligand-occupied (by ATRA and synthetic retinoids; Chaudhuri *et al.*, 1999 ▸) and non-occupied states using both mutants (Chen *et al.*, 1998 ▸) and the wild-type protein (Vaezeslami *et al.*, 2006 ▸). This cache of structural knowledge has allowed the determination of the nuclear localization sequence, as discussed previously (Sessler & Noy, 2005 ▸), as well as the development of a protein-engineering scheme to create a pH biosensor (Berbasova *et al.*, 2013 ▸).

ATRA has been in use therapeutically since the 1980s (Kligman *et al.*, 1984 ▸), but stability issues (UV photosensitivity and a proclivity for isomerization in solution) and a wide variety of off-target side effects (Tallman, 2002 ▸) have limited its impact for the treatment of complex neurological and oncological disorders. Conversely, the development of stable, activity-modifying retinoid derivatives (Chisholm *et al.*, 2016 ▸, 2019 ▸; Chisholm & Whiting, 2020 ▸; Haffez *et al.*, 2017 ▸) is now providing a greater understanding of how retinoid signalling pathways are controlled and how intervention could potentially improve the outlook for patients suffering diseases that could be challenged through retinoid intervention. Small, retinoid-like molecules are becoming increasingly important not only as drug candidates but also as part of the drug-screening process, with fluorescence-based assays and other high-throughput methodologies using their interactions with CRABPII (and other proteins) to discover and characterize the binding of small molecules from vast libraries of potential compounds (Tomlinson *et al.*, 2018 ▸; Yamada *et al.*, 2019 ▸; Tomlinson & Whiting, 2020 ▸). Understanding the structural impact of these molecules and how they may influence retinoid-related signalling outcomes through CRABPII–RAR interaction, as well as moderating nongenomic signalling effects through CRABPI, is a key step in the development of new potentially life-altering treatments.

## 2. Methods   

### 2.1. Expression of CRABPII   

GST-tagged CRAPBII protein was expressed in *Escherichia coli* BL21 (DE3) competent cells (New England Biolabs) transformed with a pGEX4T1-hCRABPII vector (canonical sequence UniProt P29373) optimized for expression in *E. coli*. Expression was started from 25 ml starter cultures (25 g l^−1^ LB broth, 100 µg ml^−1^ ampicillin), which were transferred to 1 l cultures after overnight incubation at 37°C. Expression was induced after 4 h of shaking (37°C, 150 rev min^−1^) with isopropyl β-d-1-thiogalactopyranoside (IPTG; final concentration of 1 m*M* in the culture) before shaking overnight for 20 h. The resulting cultures were pelleted using an Avanti Hi-Speed centrifuge (JLA 8.1000, 4000 rev min^−1^, 25 min, 4°C) before removal of the supernatant and freezing at −80 °C.

### 2.2. Purification of CRABPII   

Defrosted cell pellets were resuspended in 15 ml cold lysis buffer [10 m*M* DTT, 100 m*M* MgCl_2_ in phosphate-buffered saline (PBS) pH 7.5] and lysed with the addition of 0.5 mg ml^−1^ lysozyme for 30 min. The resulting suspension was sonicated on ice (2 min, 40% power) and centrifuged (20 000 rev min^−1^, 50 min, 4°C). The supernatant was loaded onto a GST affinity column (1 ml, GE Healthcare) and washed with 30 ml PBS pH 7.5. The column was then loaded with 1 ml thrombin in PBS (GE Healthcare) and kept at room temperature for 16 h to cleave the GST tag. The cleaved protein was eluted in 3 × 1 ml fractions using PBS pH 7.5 and the bound GST tag was eluted using 5 ml reduced glutathione (10 m*M* in PBS). The resulting protein fractions were analysed by SDS–PAGE and ESI mass spectrometry, and the concentration was estimated from the absorbance at 280 nm using a Thermo Scientific Nanodrop A280.

### 2.3. Expression of CRABPI-L29C   

The L29C mutant of CRABPI (CRABPI-L29C) was heterologously expressed in *E. coli* BL21 (DE3) competent cells (New England Biolabs) transformed with a pET28a-hCRABPIL29C vector (canonical sequence UniProt P29762 with an L29C modification). 25 ml starter cultures (25 g l^−1^ LB broth, 50 µg l^−1^ kanamycin) were incubated with shaking overnight (150 rev min^−1^, 37°C) and then transferred into 1 l expression flasks (LB/kanamycin) and grown with shaking at 37°C and 150 rev min^−1^. Induction with 1 ml 1 *M* IPTG (final concentration of 1 m*M* in the culture) was carried out at an OD_600_ of 0.6 before shaking overnight (20 h) at 25°C. The resulting cultures were pelleted using an Avanti Hi-Speed centrifuge (JLA 8.1000, 4000 rev min^−1^, 25 min, 4°C) before the supernatant was removed and the bacterial pellet frozen at −80°C.

### 2.4. Purification of CRABPI-L29C   

CRABPI-L29C pellets were resuspended in 15 ml loading buffer (10 m*M* imidazole, 500 m*M* NaCl, 20 m*M* Tris–HCl pH 8). The resulting suspension was sonicated on ice (40% power, 2 min) and centrifuged (Avanti Hi-Speed JA25.50, 20 000 rev min^−1^, 50 min, 4°C). The supernatant was loaded onto a HisTrap HP column (1 ml, GE Healthcare) and washed using 30 ml loading buffer. Elution was carried out in a smooth gradient with elution buffer (500 m*M* imidazole, 500 m*M* NaCl, 20 m*M* Tris–HCl pH 8) on an ÄKTA pure FPLC. The resulting protein fractions were analysed by SDS–PAGE and the concentration was estimated using a Thermo Scientific Nanodrop A280. CRABPI-L29C was incubated overnight with thrombin (∼80 U) to cleave the His tag and dialysed into fresh buffer without imidazole (500 m*M* NaCl, 20 m*M* Tris–HCl pH 8). The results of this digest were loaded onto a HisTrap HP column (1 ml, GE Healthcare) and the cleaved protein was eluted immediately using 3 ml loading buffer into 3 × 1 ml fractions. The bound tag was then eluted with elution buffer and the column was washed to remove any other contaminants. The resulting protein and tag fractions were analysed by SDS–PAGE and Nanodrop A280 before use in assays and crystallization experiments.

### 2.5. Analytical size-exclusion chromatography of CRABPI-L29C   

CRABPI-L29C was analysed for dimerization following the purification stages. 1 ml protein solution (6.8 mg ml^−1^, 500 m*M* NaCl, 20 m*M* Tris–HCl pH 8) was loaded onto a HiLoad 16/600 Superdex 75 pg column equilibrated in the same buffer using an ÄKTA pure FPLC. Elution was carried out at 1 ml min^−1^ over 3 h (1.5 column volumes in total) and fractionated into 2 ml fractions. The fractions making up the two key peaks were analysed by SDS–PAGE to demonstrate purity and protein mass, and the fractionation volumes were compared with a standard curve prepared previously. One peak consisted of monomeric CRABPI-L29C and the other corresponded by mass to a dimeric form of the protein. The resulting fractions were spun down and their concentrations were calculated to determine the fraction of dimerization.

### 2.6. Crystallization   

Diffraction-grade crystals were grown by screening conditions from commercially available crystallization kits. Purified proteins were combined with the co-crystallized retinoid (dissolved in ethanol) in a 1:1 equimolar fashion, ensuring that the overall ethanol content remained low. Screening was carried out using a Mosquito Xtal3 robot (SPT Labtech) in sitting-drop experiments (100 + 100 and 200 + 100 nl protein + reservoir drop sizes) and was followed up by optimization in sitting-drop experiments (400 µl reservoir solution) with varying drop sizes (1 + 1 and 2 + 1 µl protein + reservoir solution). The resulting crystals were inspected with a microscope and were mounted for diffraction using standard UniPuck-style pins before flash-cooling in liquid N_2_ (Teng, 1990 ▸).

CRABPI-L29C in complex with myristic acid (CRABPI-L29C–MYR) was crystallized using 0.1 *M* sodium citrate pH 5.5, 30% PEG 6000. CRABPI-L29C–DC645 was crystallized using 0.1 *M* sodium acetate pH 5.0, 25% PEG 6000 based on the optimization of a condition from PACT *premier* (Newman *et al.*, 2005 ▸) determined by robotic screening.

CRABPII–DC479 was crystallized using 0.1 *M* Tris–HCl pH 8.0, 0.2 *M* sodium sulfate, 25% PEG 4000. CRABPII–DC645 was crystallized using 0.1 *M* sodium citrate pH 5.4, 0.2 *M* ammonium acetate, 30% PEG 3350.

### 2.7. Diffraction experiments   

Diffraction experiments were carried out at Diamond Light Source (DLS) as part of the BAG program. Beamline I24 was used to collect data for CRABPII–DC645, beamline I03 was used for data collection for CRABPI-L29C–MYR and CRABPII–DC479, and beamline I04 was used for data collection for CRABPI-L29C–DC645. Further details are summarized in Table 1 ▸.

### 2.8. Data processing   

Data processing was carried out using the *CCP*4 suite of programs as well as *XDS* (Kabsch, 2010 ▸) as part of the autoprocessing performed at DLS. Scaling was carried out with *AIMLESS*/*POINTLESS* (Evans & Murshudov, 2013 ▸), phasing was performed via molecular replacement with *Phaser* (McCoy *et al.*, 2007 ▸) and manual modifications to the model were made with *Coot* (Emsley *et al.*, 2010 ▸). *REFMAC*5 was used for refinement (Murshudov *et al.*, 1997 ▸, 1999 ▸, 2011 ▸; Vagin *et al.*, 2004 ▸; Winn *et al.*, 2003 ▸; Nicholls *et al.*, 2012 ▸).

In the case of CRABPI-L29C, disulfide-bond restraints were added using SSBOND comments to suitably refine the interaction of Cys29 of chain *A* with Cys29 of chain *B* of the symmetry mate using *REFMAC*5.

In the CRABPI-L29C structures and that of CRABPII–DC479, local NCS restraints generated by *REFMAC*5 were used to improve the refinement of the structures containing a dimer or two-monomer unit (Murshudov *et al.*, 2011 ▸; Usón *et al.*, 1999 ▸). Refinement statistics are given in Table 2 ▸.

Alignments and figure production were carried out using *CCP*4*mg* (McNicholas *et al.*, 2011 ▸).

### 2.9. Binding assays of CRABPI-L29C and CRABPII   

Solutions of DC271 (300 n*M* in <1% ethanol), CRABPII (300 n*M* in PBS) and CRABPI-L29C (300 n*M* in 20 m*M* HEPES, 300 m*M* NaCl pH 7.5) and a dilution series of DC645 (9.6 µ*M* to 4.68 n*M* in <1% ethanol) were prepared. 50 µl volumes were combined in a Corning nonbinding-surface black fluorescence plate using H_2_O in place of retinoids and PBS/HEPES buffer in place of CRABPII/CRABPI-L29C when necessary for controls. The plate was spun for 2 min (1500 rev min^−1^) to ensure incorporation and the fluorescence was read with excitation at 335 nm and emission at 440 nm using a Synergy H4 plate reader. The total volume per well was 150 µl and the final concentration of protein and DC271 was 100 n*M*. The assay was adapted from Tomlinson *et al.* (2018 ▸), with additional control for intrinsic fluorescence of DC645.

## 3. Results   

### 3.1. Crystal structure of CRABPI-L29C–MYR   

CRABPI was first crystallized and its crystal structure reported in 1994 (Kleywegt *et al.*, 1994 ▸) using the proteins from *Mus musculus* and *Bos taurus*, and current PDB depositions extend to 2.7 Å resolution (PDB entry 1cbi; Thompson *et al.*, 1995 ▸). As part of crystal-screening efforts, we identified that an L29C mutation in *Homo sapiens* CRABPI allowed vastly improved crystallization and diffraction to atomic resolution, which was subsequently determined to be due to partial dimerization at the mutated surface residue. This mutation is found on the outer edge of the cap helices and as such does not affect the make-up of the hydrophobic binding site.

**Figure 1:**
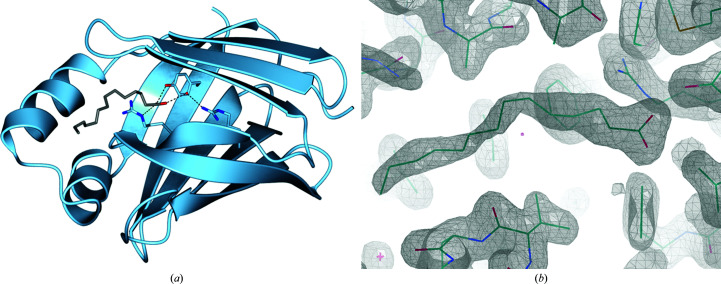
(*a*) CRABPI-L29C with the binding triad (Arg112-Arg132-Tyr134) (ribbon representation, pale blue; O atoms, red; N atoms, blue) and a conserved water molecule hydrogen-bonded to the associated ligand modelled as the 14-carbon myristic acid (stick representation, grey; O atoms, red; N atoms, blue). The protein forms a dimer with noncrystallographic symmetry which, for clarity, has been omitted. Chain *A* is displayed, with an r.m.s.d. on C^α^ atoms to chain *B* of 0.53 Å. (*b*) Ligand density of MYR in chain *A* in the ligand-binding site of CRABPI-L29C (2*F*_o_ − *F*_c_ map including the ligand in the calculation, at contour σ = 1).

As can be seen in Fig. 1 ▸, the structure (determined at a resolution of 1.64 Å) retains an overall architecture identical to that of wild-type CRABPI (r.m.s.d. of 0.69 Å to PDB entry 1cbr on C^α^ atoms) in a stable crystal form consisting of two molecules that share noncrystallographic symmetry (r.m.s.d. of 0.53 Å between chains *A* and *B* on C^α^ atoms) in the asymmetric unit, each separately dimerized with a symmetry mate. This dimerization was characterized by size-exclusion chromatography (Superdex 75 16/600) and shown to affect approximately one third of the purified protein, and is likely to exist in equilibrium with the monomeric state. This was corroborated by the presence of a dual conformation for residue Cys29 in the second monomer that can be seen in the electron density, only one version of which corresponds to a disulfide bond of 2.1 Å. The crystal structure resulting from molecular replacement contained unknown density in the ligand-binding sites of both independent monomers that was not suitable for fitting either ATRA or the synthetic ligands used for co-crystallization. It was determined that this density was most likely to represent fatty-acid byproducts from expression that were incorporated into the binding site based on their hydrophobic structure and carboxylic acid head group, and it was therefore fitted as the 14-carbon myristic acid (MYR) and the 13-carbon tridecanoic acid (TDA) in the two monomers. CRABPI shares high sequence similarity with other members of the fatty acid-binding protein family and so the incorporation of MYR/TDA molecules is a logical step for a protein produced recombinantly in *E. coli* in the absence of its natural ligand. Similarly, it has been shown that RXRs and other retinoid-binding proteins treat fatty acids (including myristic acid) as ligands, and so it is possible that nongenomic pathways are similarly influenced (Goldstein *et al.*, 2003 ▸; de Urquiza *et al.*, 2000 ▸).

### 3.2. Crystal structure of CRABPI-L29C–DC645   

**Figure 2:**
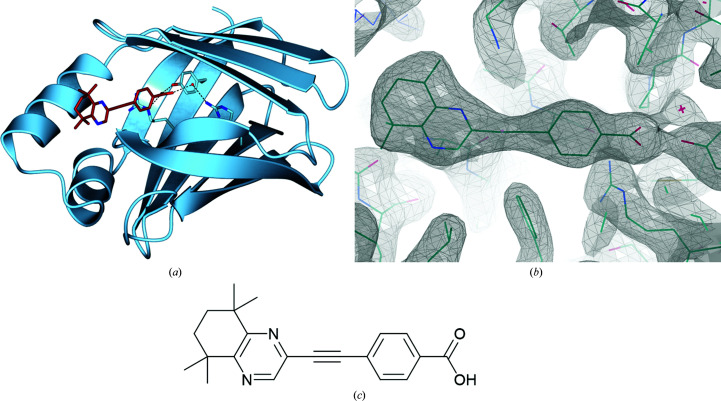
(*a*) CRABPI-L29C with the binding triad (Arg112-Arg132-Tyr134) (ribbon representation, pale blue; O atoms, red; N atoms, blue) and a conserved water molecule hydrogen-bonded to the associated DC645 ligand (brick red; O atoms, red; N atoms, blue). The asymmetric unit is composed of two independent monomers; one ligand-bound (chain *A*, shown) and one with an unoccupied binding site (the r.m.s.d. between chains *A* and *B* on C^α^ atoms is 0.64 Å). (*b*) Ligand density of DC645 in chain *A* in the ligand-binding site of CRABPI-L29C (2*F*_o_ − *F*_c_ map including ligand, contour σ = 1.00). (*c*) Chemical structure of DC645.

After the determination of the initial structure, further crystallization screening was carried out using an equimolar mixture of protein and DC645, with the aim of proper inclusion of the synthetic ligand, resulting in the structure shown in Fig. 2 ▸. Also based on CRABPI-L29C, this structure was solved at 2.41 Å resolution and contained a similar dimer, with one unoccupied binding site and one site incorporating the DC645 ligand; the r.m.s.d. between the two sites, based on C^α^ atoms, is 0.66 Å. As in the MYR-containing structure, the asymmetric unit contains a pair of molecules related by noncrystallographic symmetry; whilst one site is occupied by ligand, it is notable that the empty binding site is truly empty, *i.e.* there is no electron density evident that would suggest the incorporation of any alternate ligand. As the two monomers otherwise adopt the same overall structure, there is no immediately apparent reason for this difference. Similarly to CRABPI-L29–MYR, CRABPI-L29C–DC645 dimerization can be seen to occur across the noncrystallographic symmetry boundary, with Cys29 in both the *A* and *B* chains structured correctly to form the disulfide bridge, with no evidence that any other conformation might be occurring. It can be seen that the ligand occupies the binding site in a similar mode to the natural ligand ATRA, with the carboxylic acid group buried deep in the hydrophobic binding site adjacent to the binding triad. The lack of structural rearrangement in the protein after binding points to a more nuanced method for the involvement of CRABPI in nongenomic signalling and retinoid buffering, and is discussed in further detail below.

### 3.3. Crystal structure of CRABPII–DC479   

Wild-type CRABPII expressed recombinantly in *E. coli* proved to be reliable for crystallization in a repeatable manner and accepted ligands for co-crystallization using an equimolar combining step prior to setting up crystallization drops. Seeking to identify ligands with unconventional binding-site interactions with CRABPII, DC479 was chosen from a number of small-molecule retinoids designed to substantially improve upon the intrinsic fluorescence of the retinoid family and particularly for use in high-throughput binding assays (Chisholm *et al.*, 2019 ▸; Chisholm & Whiting, 2020 ▸). Of particular interest was the extended carbon chain to which the carboxylic acid group was appended, improving the flexibility around the head group, to determine how ligand flexibility would affect optimum binding into the triad site. The compound binds to CRABPII with an affinity in the 50 n*M* range, similar to those of other ‘strongly binding’ synthetic derivatives, and thus was an ideal choice to explore ligand-conformation space in terms of interactions with the protein (Chisholm *et al.*, 2019 ▸). Additionally, DC479 has been shown to cause the activation of nongenomic pathways whilst having no effect on genomic signalling; a result indicated by a lack of neurite outgrowth in tests with *in vitro* models (Khatib *et al.*, 2020 ▸). Indeed, its interactions with CRABPII may indicate why this is the case if the effect is caused by carrier-protein interactions rather than RAR binding (or a lack thereof) in the nucleus.

**Figure 3:**
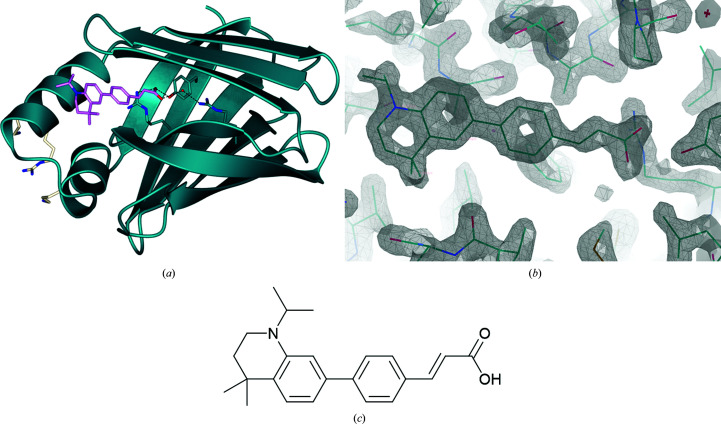
(*a*) DC479 (depicted in stick representation, pink) incorporated into the binding site of CRABPII (ribbon representation, dark blue; O atoms, red; N atoms, blue). The key binding resides (Arg112-Arg133-Tyr135) and a conserved water molecule can be seen at the base of the hydrophobic pocket with predicted hydrogen bonds to the ligand (dark blue; O atoms, red; N atoms, blue). NLS-forming residues (white; N atoms, blue) can be seen facing externally on the left. The protein forms a dimer with noncrystallographic symmetry, of which chain *A* is displayed for clarity. The r.m.s.d. between chains *A* and *B* on C^α^ atoms is 0.23 Å. (*b*) Ligand density of DC479 in chain *A* in the ligand-binding site of CRABPI (2*F*_o_ − *F*_c_ map including ligand, contour σ = 1.50). (*c*) Chemical structure of DC479.

The structure presented here (1.80 Å resolution; Fig. 3 ▸) agrees closely with previously determined CRABPII structures, including PDB entry 5ogb (C^α^ r.m.s.d. of 0.61 Å; Chisholm *et al.*, 2019 ▸), which was used for molecular replace­ment. DC479 occupies the binding site fully, with its carboxylic acid head buried adjacent to the canonical binding triad and conserved water molecule. Correct incorporation of the ligand suggests that it is not interaction with CRABPII that prevents genomic signalling in the case of DC479, which is discussed further below.

### 3.4. Crystal structure of CRABPII–DC645   

DC645 was developed as part of a library of synthetic retinoids following the EC23 archetype but lacking the fluorescence-enhancing elements used in earlier series (including DC479). Instead, a tetrahydroquinoxaline derivative offers enhanced hydrogen-bonding opportunities in the mid-section, creating interactions with solvent and Arg60 at the mouth of the binding site, which is likely to help to stabilize the protein–ligand complex. DC645 was chosen for co-crystallization owing to the increasing evidence of its positive effect on neurite outgrowth in cellular models of Alzheimer’s disease (Khatib *et al.*, 2020 ▸).

**Figure 4:**
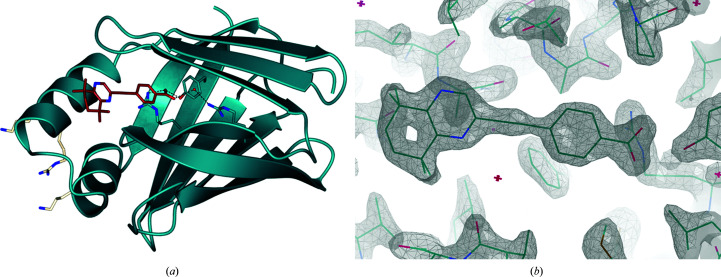
DC645 (brick red; O atoms, red; N atoms, blue) incorporated into the binding site of CRABPII (ribbon representation, dark blue; O atoms, red; N atoms, blue). The key binding resides Arg112-Arg133-Tyr135 and a conserved water molecule (dark blue; O atoms, red; N atoms, blue) can be seen at the base of the hydrophobic pocket with predicted hydrogen bonding. NLS-forming residues (white; N atoms, blue) can be seen facing externally on the left. (*b*) Ligand density of DC645 in chain *A* in the ligand-binding site of CRABPII (2*F*_o_ − *F*_c_ map including ligand, contour σ = 2.00).

The structure shown in Fig. 4 ▸, at a resolution of 1.71 Å, consists of a single CRABPII monomer with a near-identical structure to previous depositions (PDB entry 5ogb; r.m.s.d. of 0.73 Å on C^α^ atoms). The incorporation of DC645 into the CRABPII binding site, fully engaged with the binding triad, is an important step in understanding its interaction with the retinoid signalling pathway and indicates that the molecule will be transported to the nucleus.

### 3.5. Competitive binding assays   

**Figure 5:**
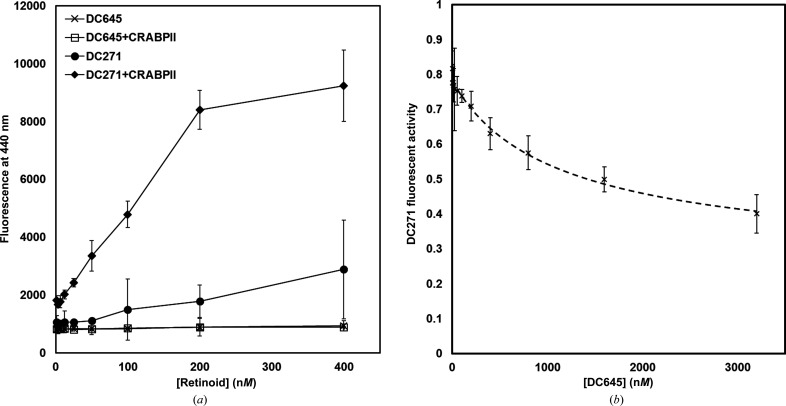
(*a*) Analysis of retinoid fluorescence of DC271 and DC645 in the presence and absence of CRABPII (*n* = 3). It can be seen that DC271 presents a stark solvatochromic effect when in the presence of CRABPII owing to inclusion into the binding site, as discussed previously (Chisholm *et al.*, 2019 ▸). DC645 conversely displays some intrinsic fluorescence but no solvatochromic effect. (*a*) Binding analysis of DC645 to CRABPII by the displacement of DC271; curve fitting was performed using *DynaFit* (Kuzmič, 1996 ▸; Tomlinson *et al.*, 2018 ▸).

To fully characterize the interaction between the protein and the ligand, the binding of DC645 to CRABPII and CRABPI-L29C was assessed using a previously published assay technique (Tomlinson *et al.*, 2018 ▸). By interpreting the displacement of a solvatochromic, fluorescent (and therefore easily quantified) retinoid from the binding site of CRABPII, the relative binding affinity of a second molecule, which would otherwise be hard to detect, can be determined. Minor modifications were made to the controls to allow for the fact that DC645 displays some small intrinsic fluorescence at the typical emission wavelength of 440 nm, which is likely to be the tail of a larger fluorescence in the sub-400 nm region. It was also demonstrated that DC645 was not solvatochromic with respect to CRABPII (Fig. 5 ▸ *a*), *i.e.* the absolute fluorescence of the molecule in solution was not dependent on the CRABPII concentration and therefore was not dependent on incorporation into the binding site. This allowed the assay to be undertaken as normal, with background controls accounting for the low-level DC645 fluorescence. Using *DynaFit* to fit the data (Fig. 5 ▸ *b*), a *K*_d_ value of 0.25 ± 0.06 µ*M* was determined for the binding of DC645 to CRABPII (Kuzmič, 1996 ▸).

**Figure 6:**
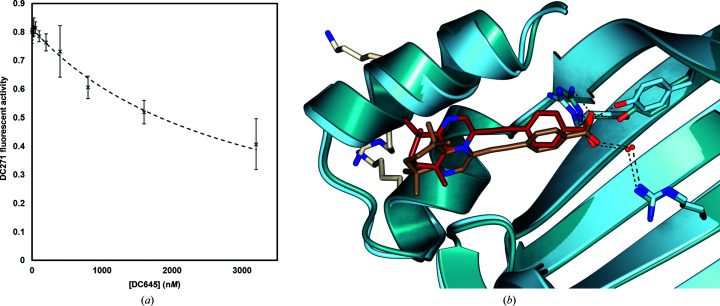
(*a*) Competitive displacement of DC271 from CRABPI-L29C by DC645 was carried out using a previously discussed methodology; curve fitting was carried out by least-squares regression using *DynaFit* (*n* = 6, α = 0.05; Tomlinson *et al.*, 2018 ▸; Kuzmič, 1996 ▸). (*b*) Superposition of CRABPII-DC645 (ribbon representation, dark blue; O atoms, red; N atoms, blue) and CRABPI-L29C–DC645 (ribbon representation, pale blue; O atoms, red; N atoms, blue) cut away to show the similarity of the ligand pose (cylinder representation; CRABPII–DC645, brick red; CRABPI-L29C–DC645, brown). The CRABPII nuclear localization sequence is shown (cylinder representation, white; N atoms, blue). The r.m.s.d. on C^α^ atoms is 0.81 Å.

Similar competitive analysis (Fig. 8) showed that CRABPI-L29C interacts less favourably with DC645, with a *K*_d_ value of 1.94 ± 0.11 µ*M* being determined for this pair. This may explain why only one of the two ligand-binding sites in the crystal structure is ligand-occupied, with the interaction being considerably weaker than in other retinoid/binding-protein pairs. For comparison of ligand-binding poses, least-squares superposition on C^α^ atoms was carried out using *CCP*4*mg* (Mc­Nicholas *et al.*, 2011 ▸), giving an r.m.s.d. between the two structures of 0.81 Å (Fig. 6 ▸ *b*). The ligand-binding residues and the carboxylic acid motif of the ligand are very closely aligned, with a slight deviation of the tetrahydroquinoxaline tail groups between the CRABPI-L29C and CRABPII structures. The slight relative translation of the molecule between the two structures is owing to the internal geometry of the binding site, which, although closely structurally conserved, does contain some modifications.

## 4. Discussion   

CRABPI and CRABPII adopt closely related tertiary structures consisting of a pair of orthogonal five-stranded β-sheets capped by two α-helices. This structure creates a hydrophobic binding pocket suitably sized for ATRA, with a triad of binding residues at its core and a swathe of hydrophobic side chains lining the pocket. This canonical structure can be considered to be the active and normal form of the protein and is adopted in all of the ligand-bound structures thus far obtained in this project.

### 4.1. Effect of the binding of DC645 on the CRABPII NLS and comparison to ATRA   

DC645 is amongst a number of retinoid-based drug candidates that have been proposed for a suite of neurodegenerative diseases, including ALS and Alzheimer’s disease. These RAR-modulating (RAR-M) compounds have been shown to diversely induce neurotrophic genes, suppress lipopolysaccharide-induced inflammation systems and promote the non-amyloidogenic pathway, suggesting the option to tailor activities for maximum effect in different pathologies (Khatib *et al.*, 2020 ▸).

**Figure 7:**
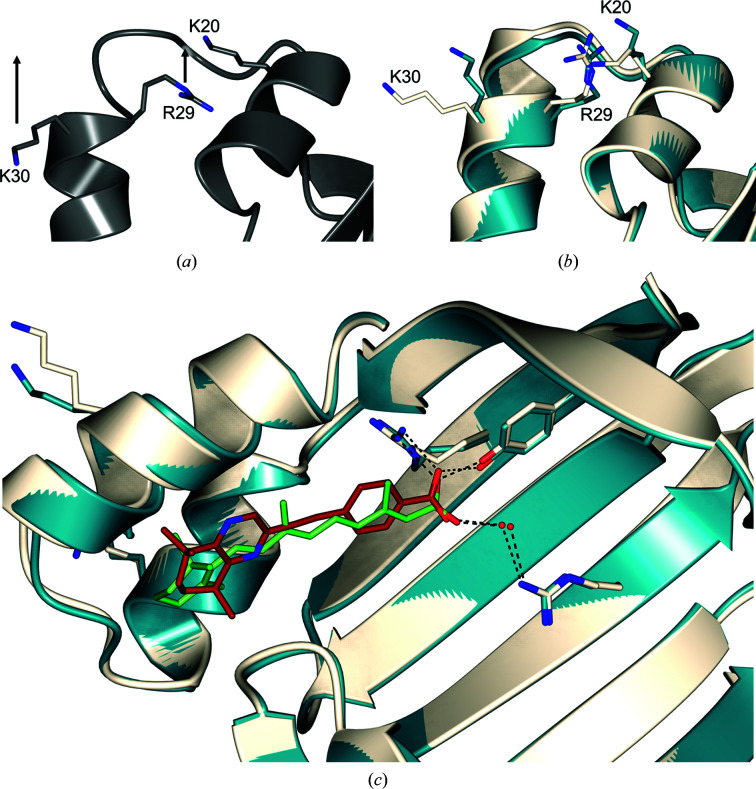
Comparison of (*a*) unoccupied CRABPII (PDB entry 1xca, grey) and (*b*) the DC645 ligand-occupied complex (blue) aligned with the ATRA ligand-occupied structure (PDB entry 1cbs, white). Arrows denote the direction of movement as residues adopt the ligand-occupied conformation. (*c*) Cut-away alignment of CRABPII–DC645 (dark blue) and PDB entry 1cbs (white), showing the similarity between the orientation of DC645 (brick red; O atoms, red; N atoms, blue) and ATRA (green; O atoms, red; N atoms, blue) and their interaction with the binding triad Arg112-Arg133-Tyr135 and a conserved water molecule. The r.m.s.d. on C^α^ atoms is 0.48 Å.

The limited flexibility of CRABPII and the lack of significant structural changes when a ligand is introduced focuses the characterization of protein–ligand interactions onto whether or not a ligand is incorporated into the binding site. Superposition of CRABPII–DC645 with an ATRA-containing structure (PDB entry 1cbs) also allows comparison between the binding position of ATRA and that of the synthetic retinoid. As shown in Fig. 7 ▸(*c*), the binding pose is near-identical and key carboxylic acid residues align closely with those of the natural ligand. Suitable conditions for hydrogen bonding can also be seen between the highly aligned binding triad Arg112-Arg133-Tyr135, the ligand and the typically conserved water molecule which serves to bridge hydrogen bonding to Arg112.

Successful ligand incorporation creates a small but notable outward inversion of Arg29, which alongside Lys20/Lys30 creates a basic region in the electrostatic surface of the protein and forms the ‘noncanonical’ nuclear localization sequence (Fig. 7 ▸). Such a shift in position suggests that a ligand will be transported through the nuclear pore and into the nucleus, allowing the opportunity to influence the signalling of both RAR and RXR proteins through the canonical pathway. This can be seen in the case of CRABPII–DC645 (Fig. 7 ▸), in which Arg29 is surface-exposed and aligned closely with the same residue as in ATRA-occupied CRABPII (PDB entry 1cbs), creating the desirable basic surface region. This residue is similarly exposed in the structure of CRABPII–DC479, suggesting that the lack of genomic signalling previously seen is not owing to a failed interaction with the carrier protein, but is rather the result of a downstream clash with the RAR/RXR signalling system. Whilst both ligands fulfil the necessary criteria for nuclear import, it is clear that corresponding *in vivo* studies are vital to fully understand the activity of RAR/RXR in the nucleus.

### 4.2. CRABPI–L29C ligand interaction and nongenomic mode of action   

**Figure 8:**
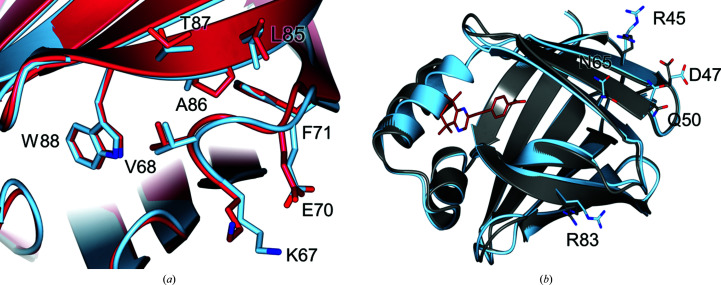
(*a*) Least-squares superposition of *H. sapiens* CRABPI-L29C–DC645 (chain *A*, pale blue) and *M. musculus* CRABPI (PSB entry 1cbr, crimson), highlighting the β-strand region residues 70–90 where the key P86A modification that differentiates the proteins is found. The r.m.s.d of the two structures on C^α^ atoms is 0.69 Å. (*b*) Alignment of unoccupied (chain *B*, grey) and ligand-occupied (chain *A*, pale blue) monomers of CRABPI-L29C–DC645, showing differences between the two forms (r.m.s.d. of 0.66 Å on C^α^ atoms).

To date, CRABPI had only been crystallized using the proteins from *M. musculus* and *B. taurus*. Whilst they are nearly identical, *H. sapiens* CRABPI contains a single amino-acid shift from alanine to proline at position 86. This single variation can be found in the lower β-sheet region proposed to be the site of interaction for Raf kinases related to non­genomic activity pathways (Park *et al.*, 2019 ▸). It is evident from alignment with PDB entry 1cbr (Fig. 8 ▸ *a*) that this mutation has no great effect upon either the location or the orientation of any local residues and has no overall impact on the general structure (Kleywegt *et al.*, 1994 ▸). This suggests that work modelled on CRABPI from *M. musculus* is relevant to the activity of the human protein.

The incorporation of a ligand into the binding site of CRABPI is currently the best available structural indicator of ligand efficacy. NMR-based studies have suggested that interactions between CRABPI and the Raf kinase involved in nongenomic pathway signalling are made at the base of the β-sandwich: the opposite end to the ligand-binding pocket (Park *et al.*, 2019 ▸). Whilst no specific sites or residues are indicated, the data suggest a method of allosteric interaction that has not yet been fully characterized. It can be seen in the structure of CRABPI-L29C–DC645 (Fig. 8 ▸ *b*) that there are several residues that adopt alternate side-chain conformations after ligand binding (aligning the ligand-occupied *A* chain with the unoccupied *B* chain) but that no overall structural rearrangement is noted beyond a slight constriction of the binding pocket. It should be noted that these residues fall within the region through which dimerization occurs, which may either contribute to their alternate conformations or be a direct result of it, allowing unoccupied/ligand-occupied cross-dimerization to occur. It is also of interest that Arg45, Asp47, Gln50 and Arg83 are amongst the few residues found in CRABPI that differ relative to CRABPII. Without further study of the specific interactions governing nongenomic signalling of CRABPI, it is difficult to assign a role to any specific residue or area of the protein, but nevertheless the high-resolution structural determination of CRABPI in the presence and absence of ligands will assist in the future development of this understanding.

The structures presented above may also offer some insight into why CRABPII binds DC645 with greater efficacy than CRABPI-L29C. It can be seen in the ligand-binding site that residue Arg60, which is positioned to create a hydrogen-bonding network between the ligand and several water molecules in CRABPII, does not have the same orientation relative to the ligand in CRABPI-L29C. It could be this small interaction that makes the binding far more favourable in the nuclear transport protein in comparison to its cytoplasm-bound cousin.

These high-resolution structures of CRABPI provide insight into how the protein compares favourably to murine and bovine orthologues, as well as how ligand binding affects the protein. It can be seen that DC645 binds to CRABPI in the canonical manner, with a deeply buried head group interacting with three members of a binding triad, and that its incorporation into the protein creates subtle shifts in residues and regions that may contribute to the previously discussed non­genomic interaction pathway.

## 5. Summary and conclusions   

The confirmation that no major structural rearrangement occurs when CRABPI binds to a ligand adds credence to the idea that the inter­actions of CRABPI with relevant binding partners to modulate non­genomic activity are mediated through subtle surface effects, including those described previously. It is notable that several residues in the lower β-sandwich region adopt alternate side-chain conformations between the vacant and ligand-bound forms of the protein and could be implicated in this process based on literature data. In the case of CRABPII, it has been demonstrated that several non-ATRA retinoid derivatives can generate suitable NLS interaction sites to prompt their import into the nucleus. This confirms a role for CRABPII in the transport of newly developed drug molecules to the relevant nuclear receptors, which is a key step in creating a selective retinoid drug and potentially a novel treatment regime for the many pathologies that retinoid signalling pathways influence.

When considering the design of ligands for drug-development programs, it is important to bear in mind that any ligand that is designed to interact with CRABPII, and hence be transported to the nucleus, may also have impacts upon the nongenomic signalling of CRABPI and as a direct result introduce unexpected phenotypic changes. It has been previously demonstrated that non­genomic and genomic pathways work in harmony as part of retinoid signalling, particularly in the case of neurite outgrowth in cell cultures, where compounds capable of inducing both pathways were most successful (Khatib *et al.*, 2019 ▸). Owing to the high sequence and structural similarity between the CRAB proteins, aiming to specifically trigger CRABPI and the nongenomic signalling pathway may prove difficult without the ability to design exceptionally selective ligands. The design of such ligands will rely heavily on data from high-throughput assays and, as demonstrated above in the semi-selectivity of DC645 for CRABPII over CRABPI, the linking of structural understanding with such data as new candidate molecules are selected. Through better understanding of both pathways, and the relative interactions of ligands with their cognate proteins, it may be possible to develop the selectivity needed to meaningfully modify retinoid signalling *in vivo* at a level that distinguishes between the genomic and nongenomic pathways.

## Supplementary Material

PDB reference: CRABPI, L29C mutant, complex with myristic acid, 7a9y

PDB reference: complex with DC645, 7a9z

PDB reference: CRABPII, complex with DC479, 7aa0

PDB reference: complex with DC645, 7aa1

## Figures and Tables

**Table 1:** Data collection and processing Values in parentheses are for the outer shell.

	CRABPI-L29C–MYR	CRABPI-L29C–DC645	CRABPII–DC479	CRABPII–DC645
PDB code	7a9y	7a9z	7aa0	7aa1
Diffraction source	I03, DLS	I04, DLS	I03, DLS	I24, DLS
Wavelength (Å)	0.9763	0.9763	0.9763	0.9763
Temperature (K)	100	100	100	100
Detector	PILATUS 6M	PILATUS 6M	PILATUS 6M	PILATUS 6M
Crystal-to-detector distance (mm)	213.39	385.58	321.83	186.39
Rotation range per image (°)	0.1	0.1	0.1	0.1
Total rotation range (°)	200	360	200	180
Exposure time per image (s)	0.05	0.05	0.05	0.1
Space group	*I*222	*P*3_1_21	*P*2_1_	*P*2_1_3
*a*, *b*, *c* (Å)	36.89, 109.98, 170.34	127.54, 127.54, 52.16	39.56, 49.92, 67.37	78.56, 78.56, 78.56
α, β, γ (°)	90.0, 90.0, 90.0	90.00, 90.00, 120.00,	90.00, 96.04, 90.00	90.0, 90.0, 90.0
Mosaicity (°)	0.00	0.00	0.70	0.00
Resolution range (Å)	36.05–1.64	110.46–2.41	49.92–1.82	45.36–1.71
Total No. of reflections	81769	366266	80669	326013
No. of unique reflections	43112	19102	23023	17775
Completeness (%)	99.9 (100)	99.9 (99.3)	97.6 (96.8)	100.0 (100.0)
Multiplicity	1.9 (1.9)	19.2 (16.8)	3.5 (3.7)	18.3 (18.3)
CC_1/2_	0.998 (0.480)	0.994 (0.398)	0.996 (0.486)	0.999 (0.548)
〈*I*/σ(*I*)〉	9.8 (1.1)†	7.1 (0.5)†	10.4 (1.6)†	18.8 (1.2)†
*R*_p.i.m._	0.029 (0.559)	0.088 (1.624)	0.059 (0.504)	0.019 (0.554)
Overall *B* factor from Wilson plot (Å^2^)	26.42	47.34	26.78	31.81

† The resolution cutoff was automatically selected based on CC_1/2_ rather than outer shell *I*/σ(*I*).

**Table 2:** Structure solution and refinement Values in parentheses are for the outer shell.

	CRABPI-L29C–MYR	CRABPI-L29C–DC645	CRABPII–DC479	CRABPII–DC645
Resolution range (Å)	36.05–1.64	110.46–2.41	66.99–1.80	45.36–1.71
Completeness (%)	99.9 (100.0)	99.9 (99.3)	99.6 (99.1)	100.0 (100.0)
No. of reflections, working set	43108	19078	23016	17753
No. of reflections, test set	2049	848	1125	889
Final *R*_cryst_	0.19	0.20	0.19	0.18
Final *R*_free_	0.22	0.25	0.25	0.20
No. of non-H atoms				
Protein	2212	2158	2184	1091
Ligand	37	25	52	25
Water	154	39	158	89
Total	2403	2222	2394	1205
R.m.s. deviations				
Bonds (Å)	0.0121	0.0088	0.0083	0.0120
Angles (°)	1.699	1.559	1.547	1.745
Average *B* factors (Å^2^)				
Protein	37.11	57.51	30.19	39.21
Ligand	51.40	49.98	27.41	32.24
Water	45.79	44.85	37.43	44.93
Ramachandran plot				
Most favoured (%)	97.03	95.88	95.94	97.78
Allowed (%)	2.23	3.00	3.32	1.48
High energy (%)	0.74	1.12	0.74	0.74
